# GR3027 antagonizes GABA_A_ receptor-potentiating neurosteroids and restores spatial learning and motor coordination in rats with chronic hyperammonemia and hepatic encephalopathy

**DOI:** 10.1152/ajpgi.00073.2015

**Published:** 2015-07-02

**Authors:** Maja Johansson, Ana Agusti, Marta Llansola, Carmina Montoliu, Jessica Strömberg, Evgenya Malinina, Gianna Ragagnin, Magnus Doverskog, Torbjörn Bäckström, Vicente Felipo

**Affiliations:** ^1^Umecrine Cognition AB, Solna, Sweden;; ^2^Umeå Neurosteroid Research Center, Department of Clinical Sciences, Umeå University, Umeå, Sweden;; ^3^INCLIVA, Valencia, Spain; and; ^4^Centro Investigación Príncipe Felipe, Valencia, Spain

**Keywords:** GABA_A_ receptors, hyperammonemia, neurosteroids, hepatic encephalopathy

## Abstract

Hepatic encephalopathy (HE) is one of the primary complications of liver cirrhosis. Current treatments for HE, mainly directed to reduction of ammonia levels, are not effective enough because they cannot completely eliminate hyperammonemia and inflammation, which induce the neurological alterations. Studies in animal models show that overactivation of GABA_A_ receptors is involved in cognitive and motor impairment in HE and that reducing this activation restores these functions. We have developed a new compound, GR3027, that selectively antagonizes the enhanced activation of GABA_A_ receptors by neurosteroids such as allopregnanolone and 3α,21-dihydroxy-5α-pregnan-20-one (THDOC). This work aimed to assess whether GR3027 improves motor incoordination, spatial learning, and circadian rhythms of activity in rats with HE. GR3027 was administered subcutaneously to two main models of HE: rats with chronic hyperammonemia due to ammonia feeding and rats with portacaval shunts (PCS). Motor coordination was assessed in beam walking and spatial learning and memory in the Morris water maze and the radial maze. Circadian rhythms of ambulatory and vertical activity were also assessed. In both hyperammonemic and PCS rats, GR3027 restores motor coordination, spatial memory in the Morris water maze, and spatial learning in the radial maze. GR3027 also partially restores circadian rhythms of ambulatory and vertical activity in PCS rats. GR3027 is a novel approach to treatment of HE that would normalize neurological functions altered because of enhanced GABAergic tone, affording more complete normalization of cognitive and motor function than current treatments for HE.

several million patients with liver cirrhosis suffer from minimal hepatic encephalopathy (MHE) with psychomotor slowing, attention deficits, mild cognitive impairment, and impaired visuomotor coordination (13, 22, 44). These neurological alterations reduce the patient's quality of life and ability to perform daily life tasks and increase the risk of traffic, work, and home accidents and the number of falls and hospitalizations. MHE predisposes to overt HE with more severe symptoms and reduces life span. Early treatment of patients with HE would improve their quality of life and life span and reduce accidents, hospitalizations, and associated costs.

Current treatments for HE are mainly directed to reduction of ammonia levels with the use of nonabsorbable disaccharides or antibiotics such as neomycin or rifaximin. Probiotics are also beginning to be tested. However, these treatments are not completely effective in reducing the neurological alterations, as they cannot eliminate hyperammonemia or inflammation continuously generated by the liver disease (14, 30, 35).

Studies in animal HE models have identified cerebral mechanisms responsible for cognitive and motor alterations and brain targets modulation of which reverses cognitive and motor alterations in rats with HE, while hyperammonemia and inflammation remain present (15).

A main contributor to cognitive and motor deficits in HE is increased GABAergic tone (increased activation of GABA_A_ receptors). Increased GABAergic tone induces motor incoordination, and extracellular GABA in cerebellum correlates with motor incoordination in rats (9). α1-Containing GABA_A_ receptors are likely involved in the motor incoordination since benzodiazepines induce ataxia by enhancing activation of α1-containing GABA_A_ receptors (27). Furthermore, overactivation of GABA_A_ receptors by the agonists diazepam and muscimol or the neurosteroids allopregnanolone and 3α,21-dihydroxy-5α-pregnan-20-one (THDOC) impairs spatial learning and memory in the Morris water maze (19, 33, 34).

GABAergic tone is increased in the cerebellum of rats with chronic hyperammonemia and HE because of increased extracellular GABA and increased levels of neurosteroids acting as positive modulators of GABA_A_ receptors (allopregnanolone, THDOC) (12). Allopregnanolone is increased in the brain of cirrhotic patients who die in HE (3), and allopregnanolone and THDOC are increased in rats with hyperammonemia (12) or portacaval anastomosis (4, 5). Hence, the neurosteroid system seems to play a role in the pathophysiology of HE and is an attractive therapeutic target for treatment of HE (4, 6). Reducing GABAergic tone with the GABA_A_ receptor antagonist bicuculline or the GABA_A_ receptor negative allosteric modulator pregnenolone sulfate improves cognitive function in hyperammonemic rats (12, 17). However, these compounds would not be good therapeutic agents, pregnenolone sulfate because it does not cross the blood-brain barrier and bicuculline and finasteride because they may have serious side effects. Bicuculline has been shown to induce seizures, epilepsy, and convulsions (8, 37, 40) and may also induce cardiovascular alterations and increase blood pressure and heart rate (24). Finasteride may have adverse effects on sexual function, high-grade prostate cancer incidence, central nervous system (CNS) function, and depression (38).

Modulation of GABAergic tone by acting on the neurosteroid system could thus have more therapeutic benefits. In this line, inhibition of neurosteroid synthesis with finasteride improves symptoms and prevents hepatic coma in thioacetamide (TAA)-induced HE (28). However, the use of finasteride in patients is limited by its side effects. We have developed a new compound, GR3027, that selectively antagonizes the enhanced activation of GABA_A_ receptors by neurosteroids such as allopregnanolone and THDOC. The aims were to assess whether GR3027 improves motor incoordination and spatial learning in rats with chronic hyperammonemia or with portacaval anastomosis, two main models of HE recommended by the International Society for Hepatic Encephalopathy (10).

## MATERIALS AND METHODS

GR3027 is a 3β-hydroxysteroid within a series of molecules developed and patented by some of the authors (T. Bäckström and G. Ragagnin) for the treatment of CNS disorders (patent WO 2008/063128). 3β-Hydroxysteroids are functional antagonists against 3α-hydroxysteroids that positively modulate GABA_A_ receptors (36, 42, 43).

### GABA_A_ Receptor Pharmacology

HEK293 cells were permanently transfected to constitutively express the human α5, β3, and γ2L or α1, β2, and γ2L GABA_A_ receptor subunits, respectively. Cells were detached by trypsin, incubated for 15 min at 37°C in extracellular (EC) solution (in mM: 137 NaCl, 5.0 KCl, 1.0 CaCl_2_, 1.2 MgCl_2_, 10 HEPES, and 10 glucose, pH 7.4), and added to the EC solution in the chip bath (Dynaflow Resolve, Cellectricon).

Whole cell recordings were performed under voltage-clamp conditions. Recordings were performed at room temperature and −17 mV (corrected for liquid junction potential) with an AxonPatch 200B amplifier and a DigiData 1322A converter. Data were acquired with pCLAMP 9.0 and analyzed with Clampfit 9.0 (Axon Instruments, Foster City, CA). Patch electrodes (1.5–4 MΩ) were filled with intracellular solution (in mM: 140 Cs-gluconate, 3.0 NaCl, 1.2 MgCl_2_, 10 HEPES, 1.0 EGTA, 2 MgATP, pH 7.2).

THDOC and GR3027 were dissolved in dimethyl sulfoxide (DMSO) and diluted in EC solution; at measurements all solutions contained 0.1% DMSO.

Different protocols were used for different electrophysiology measurements, as the GABA_A_ receptor subunit combinations studied are present in different parts of neurons [recently reviewed by Carver and Reddy (11)]. As α1β2γ2L-GABA_A_ receptors are present within the synapse in vivo, experimental conditions resembling that situation, a short application (40 ms) of a high GABA concentration (30 μM), were used. In contrast, α5β3γ2L-GABA_A_ receptors are present extrasynaptically in vivo, and thus the experimental conditions used were long exposures (6 s) to a low GABA concentration (0.3 μM). With both cell types the EC_75_s of THDOC were used, i.e., 100 nM for studies of α1β2γ2L and 200 nM when α5β3γ2L-expressing cells were evaluated. Cells were preexposed to THDOC alone or to THDOC in the presence of GR3027 before the GABA application. In each cell effects were normalized to the control response and the area under the curve was analyzed.

### Off-Target Profiling

The binding of GR3027 was determined for receptors, ion channels, and enzymes, including all major classes of neurotransmitter receptors. A total of 114 targets (Table 1) were tested in duplicate with GR3027 at 10 μM (PerkinElmer, customized screen). Binding activity was defined as ≥50% inhibition of ligand binding.

**Table 1.** Targets in binding studies with GR3027

Adenosine A1 receptor	Angiotensin II, AT1 receptor
Adenosine A2A receptor	Angiotensin II, AT2 receptor
Adenosine A3 receptor	Bradykinin receptor
Adrenergic alpha 1A receptor	Cholecystokinin CCK1 receptor
Adrenergic alpha 1B receptor	Cholecystokinin CCK2 receptor
Adrenergic alpha 2A receptor	Endothelin receptor A
Adrenergic alpha 2B receptor	Endothelin receptor B
Adrenergic Alpha 2C receptor	Galanin receptor
Adrenergic beta 1 receptor	Neurokinin 1 receptor
Adrenergic beta 2 receptor	Neurokinin 2 receptor
Adrenergic beta 3 receptor	Neurokinin 3 receptor
Cannabinoid CB1 receptor	Neuropeptide Y receptor Y1
Cannabinoid CB2 receptor	Neuropeptide Y receptor Y2
Dopamine transporter	Vasoactive intestinal peptide receptor
Dopamine D1 receptor	Vasopressin receptor 1
Dopamine D2 s receptor	Calcium channel type L, BDZ site
Dopamine D3 receptor	Calcium channel type L, dihydropyridine site
Dopamine D4.4 receptor	Calcium channel type N
GABA transporter	Potassium channel, ATP-sensitive
GABA-A receptor, agonist site	Potassium channel, Ca^2+^-activated
GABA-A receptor, α1, benzodiazepine site	Potassium channel, hERG
GABA-A receptor, α6, benzodiazepine site	Sodium channel, site 2
GABA-A receptor, chloride channel	Adenylate cyclase, forskolin
GABA-B receptor	Nitric oxide synthase
Glutamate receptor, AMPA site	Protein kinase C
Glutamate receptor, kainate site	Carbonic anhydrase 1
Glutamate receptor, MK-801 site	Carbonic anhydrase 2
Glutamate receptor, NMDA agonist site	Histone deacetylase 3
Glutamate receptor, NMDA, glycine site	Histone deacetylase 6
Glycine receptor, strychnine-sensitive site	Histone deacetylase 8
Histamine H1 receptor	Histone deacetylase sirtuin 1
Histamine H2 receptor	Histone deacetylase sirtuin 2
Histamine H3 receptor	Histone deacetylase sirtuin 3
Imidazoline I2 receptor	Plasma esterase
Melatonin receptor	Monoamine oxidase A, peripheral
Translocator protein (TSPO)	Monoamine oxidase B, peripheral
Muscarinic M1 receptor	Cyclooxygenase 1
Muscarinic M2 receptor	Cyclooxygenase 2
Muscarinic M3 receptor	Serine/threonine phosphatase, PP1a
Muscarinic M4 receptor	Serine/threonine phosphatase, PP2a
Muscarinic M5 receptor	Phosphodiesterase 4A1A
Nicotinic receptor, neuronal	Phosphodiesterase 5A1
Norepinephrine transporter	Protease ACE1
Opioid receptor, delta 2	Protease elastase
Opioid receptor, kappa 1	Matrix metalloprotease 2
Opioid receptor, mu	Matrix metalloprotease 3
Opioid receptor, Orphanin, ORL1	Cyclin-dependent kinase 2
Purinergic P2Y receptor	Calcium/calmodulin-dependent kinase 2A
Serotonin receptor, nonselective	Epidermal growth factor receptor
Sigma receptor, nonselective	Ephrin type-A receptor
Estrogen receptor alpha	Inhibitor of nuclear factor kappa-B kinase, b
Glucocorticoid receptor, ligand domain	Insulin receptor
Progesterone receptor	Mitogen-activated protein kinase C
Testosterone receptor, cytosolic	Protein kinase C-alpha
Corticotropin-releasing factor receptor	Protein kinase C-eta
Platelet-activating factor receptor	Proto-oncogene tyrosine-protein kinase Src
Thyrotropin-releasing hormone receptor	Zeta-chain-associated protein kinase 70

Binding targets for studies with 10 μM GR3027 are shown.

### Chronic Hyperammonemia in Rats

Male Wistar rats (140–160 g) were made hyperammonemic by feeding with a diet containing 25% ammonium acetate (16). These rats become hyperammonemic, with two- to threefold increase in blood ammonia and ∼40% increase in the brain (7). Control rats were fed normal chow.

### Portacaval Anastomosis

Male Wistar rats (220–240 g) were subjected to portacaval anastomosis. Rats were anesthetized, and an end-to-side portacaval anastomosis was constructed under aseptic conditions with a continuous suture technique according to the technique of Lee and Fisher (21). The inferior vena cava and portal vein were clamped for not more than 15 min; after unclamping, the bowel was evaluated for cyanosis. If cyanosis persisted, the animal was killed. Control rats were sham operated. Sham-operated rats had their portal vein and inferior vena cava clamped for 10 min. After satisfactory surgery, the abdomen was sutured in two layers and rats were returned to their individual cages.

Adequate measures were taken to minimize pain and discomfort to the animals. The experiments were approved by the Comite de Experimentación y Bienestar Animal of our Center and performed in accordance with guidelines of the Directive of the European Commission (2010/63/EU) for care and management of experimental animals.

### Treatment with GR3027

GR3027 in sesame oil was administered by daily subcutaneous injections in the back. Two different sets of experiments were performed in hyperammonemic rats. In the first set four groups of rats were used: *1*) control rats injected with vehicle, *2*) hyperammonemic rats injected with vehicle, *3*) control rats injected with 20 mg/kg GR3027, and *4*) hyperammonemic rats injected with 20 mg/kg GR3027. Control rats injected with GR3027 were not included subsequently because no relevant effect was found in these rats. In the second set of experiments five groups of rats were used: *1*) control rats injected with vehicle, *2*) hyperammonemic rats injected with vehicle, and hyperammonemic rats injected with *3*) 3, *4*) 10, or *5*) 20 mg/kg GR3027. Numbers of rats are indicated in figures.

In rats GR3027 is metabolized in the liver, and GR3027 dosage adjustments were made in the portacaval shunt (PCS) rats to compensate for the metabolic dysfunction caused by the portacaval shunting. For this a prestudy with different doses was performed, and thereafter the following groups were used: *1*) sham-operated rats injected with vehicle, *2*) PCS rats injected with vehicle, and PCS rats injected with *3*) 0.7 or *4*) 2.5 mg/kg GR3027. Numbers of rats are indicated in figures. The reason that the doses used in the studies with hyperammonemic and PCS rats are different is that GR3027 is metabolized by the liver in rats and we expected a reduced metabolism of GR3027 in PCS compared with control or hyperammonemic rats. For this reason we performed prestudies with different doses of GR3027 in PCS rats to find the doses giving equal exposures in the two rat models. The doses were then reduced in the experiments with PCS rats compared with those with hyperammonemic rats. As shown in Fig. 7, the levels of GR3027 reached in plasma and brain were similar in hyperammonemic and PCS rats.

### Experimental Design

**Fig. 1.**
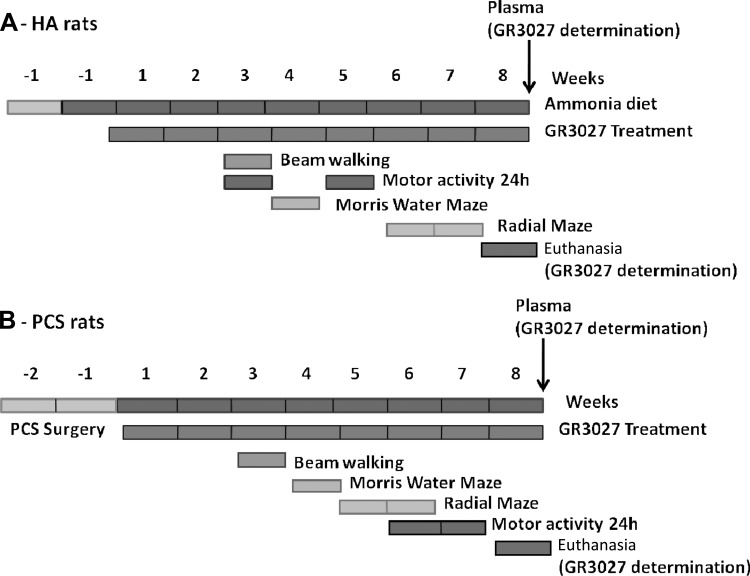
Scheme showing the experimental design for hyperammonemic (HA; *A*) rats and portacaval shunt (PCS; *B*) rats.

The experimental design, including the treatment period and the time at which the behavioral tests were conducted in the two experimental models, is shown in Fig. 1.

#### Motor coordination: beam walking test.

Motor coordination was tested as described in Gonzalez-Usano et al. (17) with a wood strip (20-mm diameter). The number of foot faults (slips) was manually recorded as a measure of incoordination.

#### Spatial learning in Morris water maze.

The test was performed as in Monfort et al. (29) with a circular pool (160 × 40 cm). After pretraining, the rats were trained to learn the fixed location of the invisible platform over 3 days. Training consisted of three swims per day. The time needed to find the hidden platform was recorded as a measure of learning. Spatial memory was assessed 24 h later by removing the platform and measuring the time spent in the quadrant where it was previously positioned.

#### Spatial learning in eight-arm radial maze.

Training was composed of three trials per day over 6 days. The task involved locating four pellets placed at the end of different arms according to a random configuration as in Hernandez-Rabaza et al. (18). The number of working memory errors (visits to arms already visited in the same trial) were recorded.

#### Circadian rhythms of locomotor activity.

Motor activity was recorded continuously at intervals of 5 min for 14 days in a 12:12-h light-dark cycle with an actimeter of infrared motion detection as in Ahabrach et al. (1). Ambulatory counts and vertical counts were recorded (1). Activity was detected by arrays of infrared motion detection, with two arrays 1 cm above the floor of the chamber and another array 6 cm above the floor. One ambulatory count is recorded when the rats interrupted three consecutive infrared detectors. One vertical count is recorded when the rats interrupted the above detectors.

#### Determination of ammonia.

Blood ammonia was measured with the II Ammonia Arkray test kit (PocketChem BA, Arkray) using 20 μl of blood.

#### GR3027 exposure.

At the end of treatment plasma was collected from the tail vein. After death by decapitation brains were collected and frozen on dry ice. For analysis of GR3027, brain tissue was homogenized with a 1-to-4 ratio of tissue to PBS (pH 7.4) and extracted with 2 volumes of methanol-acetonitrile (1:1). Plasma protein was precipitated with 3 volumes of acetonitrile. Analyses were performed with a Waters ACQUITY UPLC+Waters XEVO-TQS triple quadrupole mass spectrometer (Admescope, Oulu, Finland). For calculations of the amount of free GR3027 in the brain the fraction unbound (F_ub_) in brain homogenates was determined by dialysis: F_ub_ in hyperammonemia = 0.70% and F_ub_ in PCS = 1.43% (Admescope).

### Statistical Analysis

All data are shown as means ± SE.

#### Electrophysiology.

Each data point has its own control; *n* = number of data points pooled from 3–11 cells. Kruskal-Wallis test followed by the paired nonparametric Wilcoxon signed-ranks test (2 related samples) were used to evaluate effects of GR3027. SPSS statistical package versions PASW 19.0 and 22 were used for all statistical tests.

#### Animal data.

Statistical significance was estimated with two-way ANOVA and Bonferroni post hoc test and with Student's *t*-test when only one parameter was compared with GraphPad Prism (La Jolla, CA).

## RESULTS

### GR3027 Antagonizes THDOC but not GABA at the GABA_A_ Receptor

**Fig. 2.**
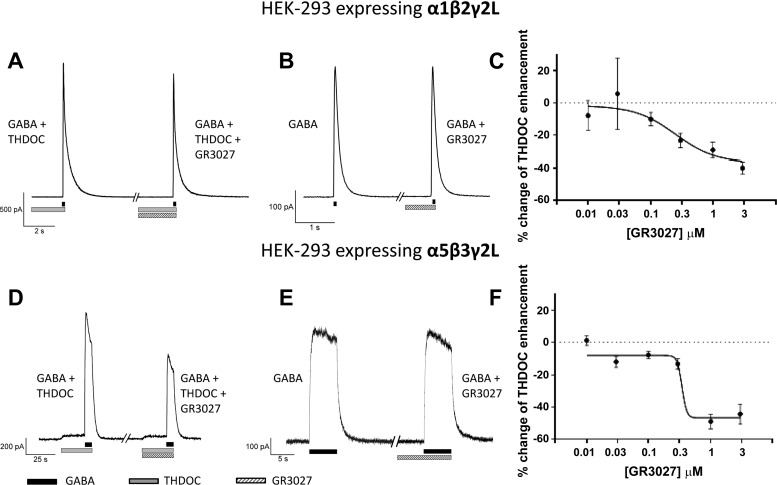
Representative patch-clamp current measurements showing GR3027 antagonism of the 3α,21-dihydroxy-5α-pregnan-20-one (THDOC)-enhanced GABA modulation of α1β2γ2L and α5β3γ2L GABA_A_ receptors and no inhibition of GABA. *A*: 1 μM GR3027 antagonism of 100 nM THDOC enhanced 30 μM GABA-mediated current response with the α1β2γ2L GABA_A_ receptor. *B*: 1 μM GR3027 did not antagonize the 30 μM GABA response of the α1β2γ2L GABA_A_ receptor. *C*: concentration response of the GR3027 antagonism of 100 nM THDOC enhanced 30 μM GABA-mediated current response with the α1β2γ2L GABA_A_ receptor. *D*: 1 μM GR3027 antagonism of 200 nM THDOC enhanced 0.3 μM GABA-mediated current response with the α5β3γ2L GABA_A_ receptor. *E*: 1 μM GR3027 did not antagonize the 0.3 μM GABA response of the α5β3γ2L GABA_A_ receptor. *F*: concentration response of the GR3027 antagonism of 200 nM THDOC enhanced 0.3 μM GABA-mediated current response with the α5β3γ2L GABA_A_ receptors.

The effects of GR3027 were studied with patch-clamp measurements on recombinant HEK293 cells expressing human variants of the GABA_A_ receptor. GR3027 (1 μM) antagonizes the effect of THDOC at both the α1β2γ2L- and α5β3γ2L-subunit variants of the GABA_A_ receptor (Fig. 2, *A* and *C*). With the α1β2γ2L receptor 1 μM GR3027 inhibits 29 ± 5% of THDOC enhancement of the GABA response (*P* < 0.001), and with the α5β3γ2L receptor the inhibition is 49 ± 5% (*P* < 0.001) in the experimental conditions used.

In contrast, GR3027 (1 μM) does not antagonize the effect of GABA at the GABA_A_ receptor (Fig. 2, *B* and *E*). There is no significant effect of GR3027 at either the α1β2γ2L GABA_A_ receptor [−3.1 ± 1.7%, not significant (NS)] or the α5β3γ2L GABA_A_ receptor (−3.8 ± 1.5%, NS) when GABA is the sole activator. GR3027 dose-dependently antagonized THDOC (Fig. 2, *C* and *F*, *P* < 0.001). During the specific conditions used for the different receptors, IC_50_ = 250 nM for the α1β2γ2L GABA_A_ receptor and 350 nM for the α5β3γ2L GABA_A_ receptor.

### Off-Target Binding Profile

At 10 μM GR3027 did not show binding activity at any of the studied neurotransmitter-related receptors, steroid receptors, or peptide receptors in Table 1.

### GR3027 Restores Motor Coordination in Hyperammonemic and PCS Rats

**Fig. 3.**
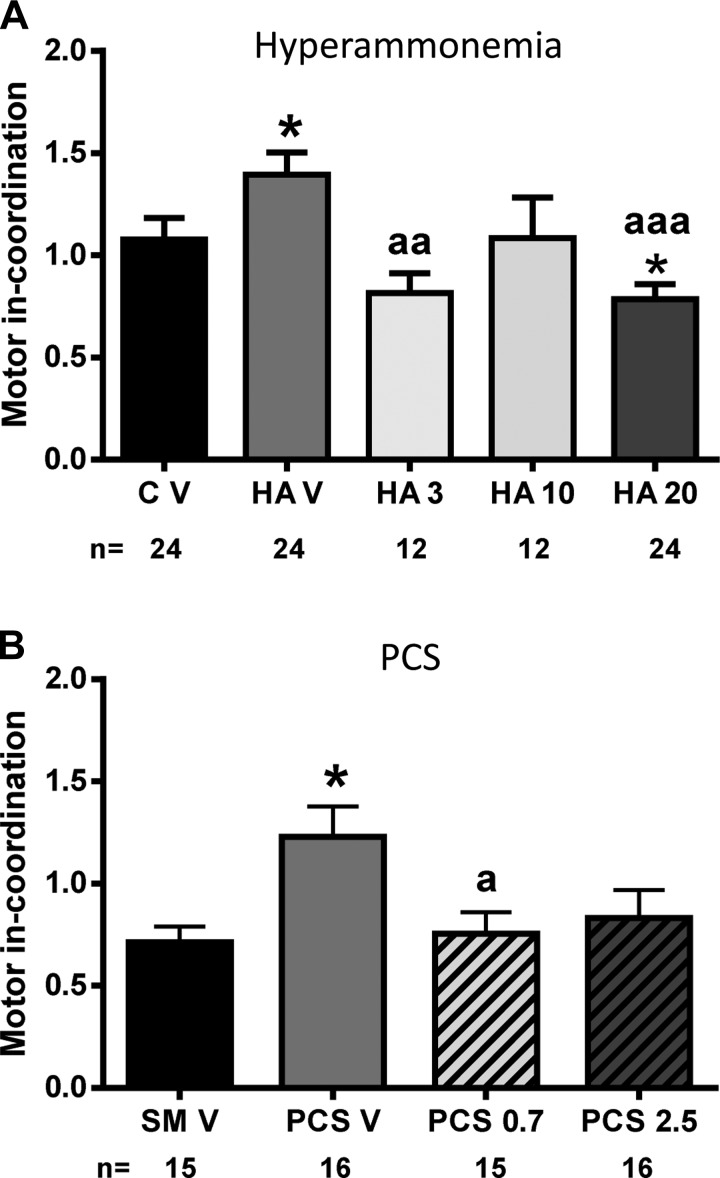
GR3027 restores motor coordination in beam walking in hyperammonemic and PCS rats. *A*: control (C V) or hyperammonemic (HA V) rats treated with vehicle and hyperammonemic rats treated with 3 (HA 3), 10 (HA 10), or 20 (HA 20) mg/kg GR3027. *B*: sham-operated control rats (SM V) or PCS rats treated with vehicle (PCS V) and PCS rats treated with 0.7 (PCS0.7) or 2.5 (PCS2.5) mg/kg GR3027. Values are means ± SE for number of rats indicated under each bar. *Different from control or sham-operated rats: *P < 0.05. Different from hyperammonemic or PCS rats: ^a^*P* < 0.05, ^aa^*P* < 0.01, ^aaa^*P* < 0.001.

Hyperammonemic rats show motor incoordination in beam walking, with more (*P* < 0.05) slips (1.4 ± 0.1) than control rats (1.0 ± 0.1). GR3027 restores motor coordination (Fig. 3*A*) at 3 mg/kg (0.8 ± 0.1 slips, *P* < 0.05) and 20 mg/kg (0.8 ± 0.1 slips, *P* < 0.05). At 10 mg/kg GR3027 also normalized motor coordination (1.0 ± 0.2 slips), but not significantly (Fig. 3*A*).

PCS rats show motor incoordination, with more (*P* < 0.01) slips (1.2 ± 0.1) than control rats (0.71 ± 0.07). GR3027 also restores motor coordination in PCS rats (Fig. 3*B*), reducing the number of slips to 0.75 ± 0.10 (*P* < 0.05 vs. PCS) for 0.7 mg/kg and to 0.8 ± 0.1 (*P* = 0.058) for 2.5 mg/kg (Fig. 3*B*).

### GR3027 Restores Spatial Memory in Morris Water Maze

**Fig. 4.**
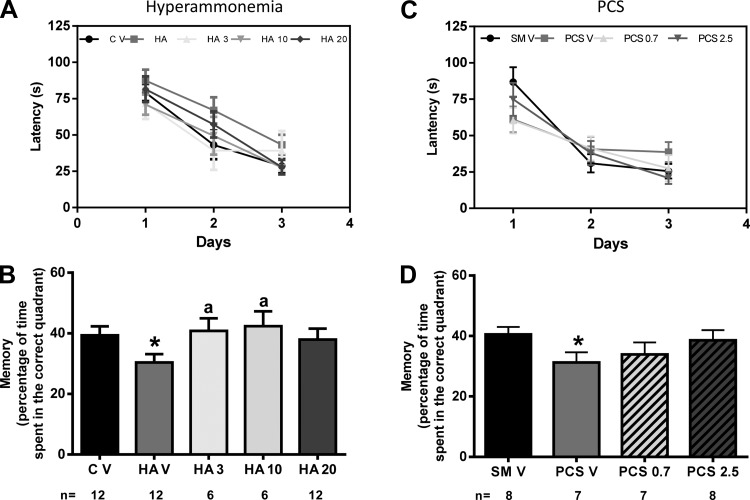
GR3027 restores spatial memory in the Morris water maze in hyperammonemic and PCS rats. Spatial learning and memory was assessed in control (C V) or hyperammonemic (HA V) rats treated with vehicle and hyperammonemic rats treated with 3 (HA 3), 10 (HA 10), or 20 (HA 20) mg/kg GR3027 (*A* and *B*) and in sham-operated control rats (SM V) or PCS rats treated with vehicle (PCS V) and PCS rats treated with 0.7 (PCS 0.7) or 2.5 (PCS 2.5) mg/kg GR3027 (*C* and *D*). *A* and *C*: escape latencies to reach the platform during learning sessions. *B* and *D*: time spent in the correct quadrant during the memory test. Values are means ± SE for the number of rats indicated under each bar. Different from control or sham rats: *P < 0.05. Different from hyperammonemic or PCS rats: ^a^*P* < 0.05.

As shown in Fig. 4, *A* and *C*, all groups of rats learned the position of the hidden platform, i.e., decreased the time to swim to the platform over the 3 days with practice. Escape latencies were slightly longer in hyperammonemic (Fig. 4*A*) and PCS (Fig. 4*C*) rats than in control rats at *day 3*, but the differences did not reach statistical significance.

Spatial memory was reduced (*P* < 0.05) in hyperammonemic rats, such that in the memory test they remained for less time in the right quadrant (30 ± 2% of time) than control rats (39 ± 2%). GR3027 restored spatial memory in the Morris water maze. The percentages of time spent in the correct quadrant were 41 ± 4%, 42 ± 5%, and 38 ± 3% for 3, 10, and 20 mg/kg doses, respectively (Fig. 4*B*).

Spatial memory was also reduced (*P* < 0.05) in PCS rats. In the memory test PCS rats remained for less time in the right quadrant (31 ± 3% of time) than control rats (41 ± 2%). GR3027 restored spatial memory. The percentages of time spent in the correct quadrant were 34 ± 4% and 39 ± 3% for 0.7 and 2.5 mg/kg doses, respectively (Fig. 4*D*).

### GR3027 Restores Spatial Learning in Radial Maze

**Fig. 5.**
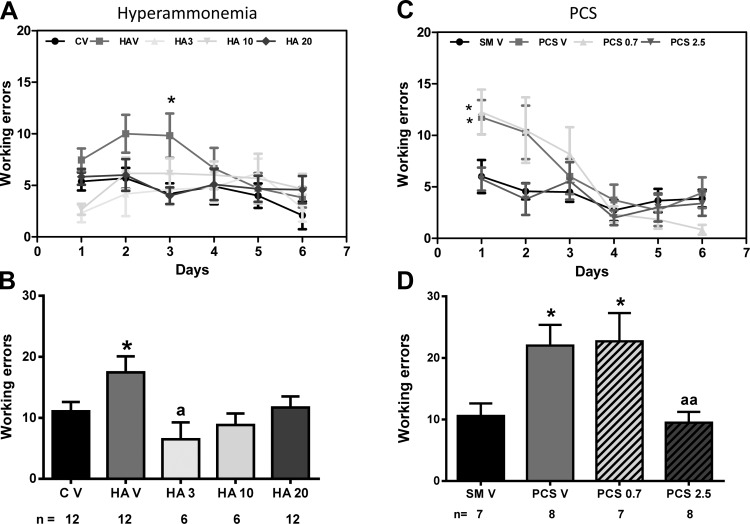
GR3027 restores spatial learning in the radial maze in hyperammonemic and PCS rats. Spatial learning in the radial maze was assessed in control (C V) or hyperammonemic (HA V) rats treated with vehicle or (for hyperammonemic rats) treated with 3 (HA 3), 10 (HA 10) or 20 (HA 20) mg/kg GR3027 (*A* and *B*), in sham-operated control rats (SM V) or PCS rats treated with vehicle (PCS V), and in PCS rats treated with 0.7 (PCS 0.7) or 2.5 (PCS 2.5) mg/kg GR3027 (*C* and *D*). *A* and *C*: working errors during the different sessions. *B* and *D*: working errors during *days 1* and *2*. Values are means ± SE for the number of rats indicated under each bar. Different from control or sham-treated rats: *P < 0.05. Different from hyperammonemic or PCS rats: ^a^*P* < 0.05, ^aa^*P* < 0.01.

Hyperammonemic rats showed reduced spatial learning in the radial maze (Fig. 5*A*). The number of working errors in *days 1* and *2* was higher (*P* < 0.05) in hyperammonemic rats (18 ± 3 errors) than in control rats (11 ± 1.5 errors; Fig. 5*B*). Hyperammonemic rats treated with GR3027 behaved as control rats. The number of errors was 6.5 ± 2.8, 8.8 ± 1.9, and 12 ± 2 for 3, 10 and 20 mg/kg doses, respectively (Fig. 5*B*).

PCS rats also showed reduced spatial learning in the radial maze (Fig. 5*C*). The number of working errors in *days 1–2* (Fig. 5*D*) was higher (*P* < 0.01) in PCS rats (22 ± 2 errors) than in sham-operated rats (10 ± 2 errors). Treatment of PCS rats with 0.7 mg/kg GR3027 was not enough to improve performance in the radial maze (23 ± 2 errors). Treatment with 2.5 mg/kg GR3027 completely normalized performance in the radial maze (11 ± 1 errors, *P* < 0.05 vs. PCS). In PCS rats, therefore, treatment with 0.7 mg/kg GR3027 is not enough to restore spatial learning, while 2.5 mg/kg restores it (Fig. 5*D*). This indicates a beneficial effect of GR3027 on spatial learning.

### GR3027 Partially Restores Circadian Rhythm of Motor Activity in PCS Rats

**Fig. 6.**
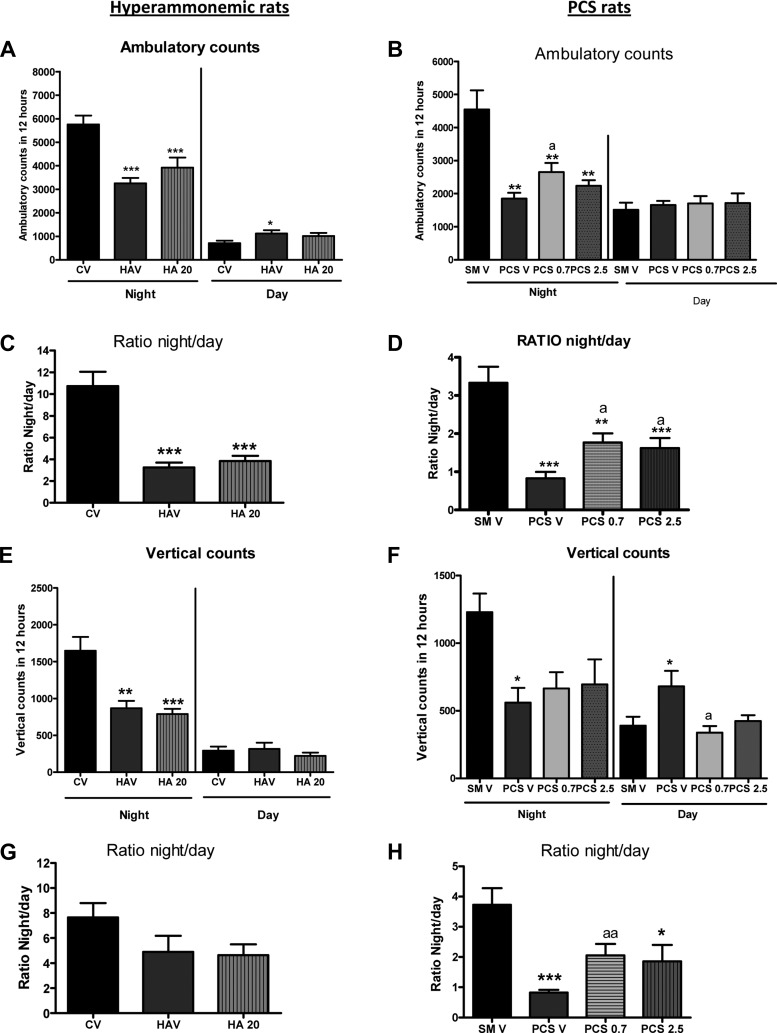
GR3027 partially restores the circadian rhythm of spontaneous motor activity. Ambulatory counts (*A–D*) and vertical activity (*E–H*) were assessed in control (C) and hyperammonemic (HA) rats (*A*, *C*, *E*, *G*) treated with vehicle (V) or with 20 mg/kg GR3027 and in sham-operated control rats (SMV) or PCS rats (*B*, *D*, *F*, *H*) treated with vehicle (PCSV) or with 0.7 (PCS0.7) or 2.5 (PCS2.5) mg/kg GR3027. Motor activity during night and day is shown in *A*, *B*, *E* and *F* and the ratios of activity night to day in *C*, *D*, *G*, and *H*. Values are means ± SE of 8 rats per group. Different from hyperammonemic or PCS rats: ^a^*P* < 0.05, ^aa^*P* < 0.01. Different from control/sham: *P < 0.05, **P < 0.01, ***P < 0.001.

PCS rats show reduced motor activity (ambulatory counts) during the night (active phase for rats), showing 1,849 ± 176 counts, which is lower (*P* < 0.05) than in control rats (4,546 ± 584 counts). GR3027 at 0.7 mg/kg increased (*P* < 0.05) the activity in PCS rats to 2,652 ± 275 counts. At 2.5 mg/kg GR3027 did not affect ambulatory counts (2,235 ± 170 counts) (Fig. 6*B*).

The night-to-day ratio of ambulatory activity was reduced in PCS rats, indicating altered circadian rhythm (Fig. 6*D*). This ratio was 3.3 ± 0.4 in control rats and was reduced (*P* < 0.001) in PCS rats to 0.8 ± 0.16. GR3027 improved (*P* < 0.05) the night-to-day ratio of activity, reaching 1.7 ± 0.2 and 1.6 ± 0.3 for 0.7 and 2.5 mg/kg, respectively. This indicates partial restoration of circadian rhythm of activity.

Hyperammonemic rats also showed reduced ambulatory activity during the night and night-to-day ratio of activity compared with control rats (Fig. 6, *A* and *C*). GR3027 at 20 mg/kg showed a tendency to slightly increase the activity and the ratio, but the effect did not reach statistical significance.

### GR3027 Normalizes Vertical Activity During the Day and Partially Restores Its Circadian Rhythm

PCS rats showed reduced vertical activity during the night (active phase), showing 561 ± 108 counts, which is lower (*P* < 0.05) than in control rats (1,228 ± 138 counts). GR3027 at 0.7 mg/kg and 2.5 mg/kg did not affect nocturnal vertical activity (664 ± 121 and 695 ± 185 counts, respectively) (Fig. 6*F*).

In contrast, PCS rats show increased vertical activity during the day, showing 682 ± 114 counts, which is higher (*P* < 0.05) than in control rats (391 ± 64 counts). GR3027 at 0.7 mg/kg and 2.5 mg/kg completely normalized vertical activity during the day, reaching 339 ± 47 and 424 ± 44 counts, respectively (Fig. 6*F*).

The night-to-day ratio of vertical activity is reduced in PCS rats, indicating altered circadian rhythm (Fig. 6*H*). This ratio is 3.7 ± 0.6 in control rats and is reduced in PCS rats to 0.8 ± 0.01 (*P* < 0.001). GR3027 improved (*P* < 0.01) the night-to-day ratio of activity, reaching 2.1 ± 0.4 and 1.9 ± 0.6 for 0.7 and 2.5 mg/kg, respectively (Fig. 6*H*). This indicates partial restoration of circadian rhythm of vertical activity.

Hyperammonemic rats also showed reduced vertical activity during the night and night-to-day ratio of activity compared with control rats (Fig. 6, *E* and *G*). GR3027 at 20 mg/kg did not affect the activity or the ratio.

### GR3027 Does Not Affect Ammonia Levels

Blood ammonia levels were increased (*P* < 0.001) in hyperammonemic rats (167 ± 16 μM) compared with control rats (47 ± 3 μM). GR3027 did not affect ammonia levels in hyperammonemic rats (139 ± 14 μM).

Blood ammonia levels were also increased (*P* < 0.001) in PCS rats (411 ± 39 μM) compared with sham-operated rats (51 ± 13 μM). GR3027 did not affect blood ammonia, which remained at 380 ± 22 and 348 ± 75 μM in PCS rats treated with 0.7 and 2.5 mg/kg GR3027, respectively.

### GR3027 Exposure in Hyperammonemic and PCS Rats

**Fig. 7.**
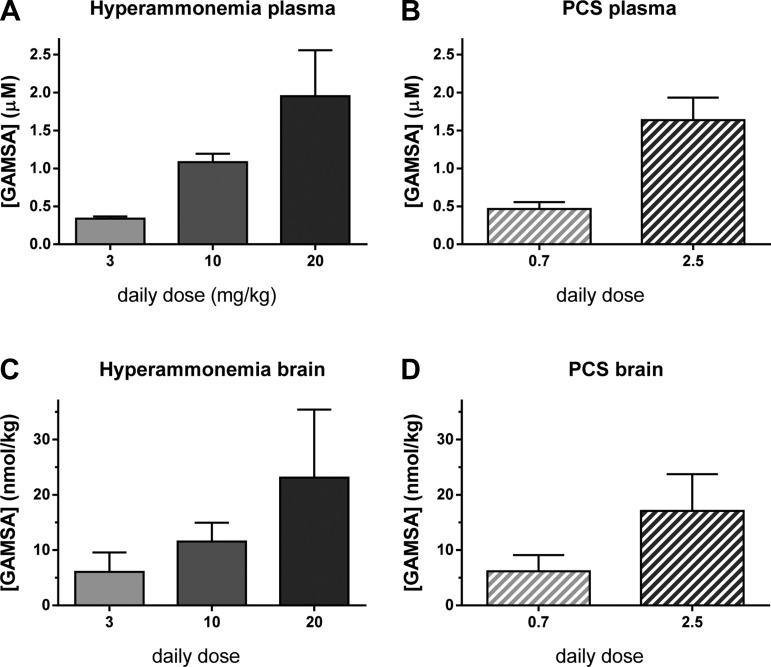
GR3027 exposures in plasma and in the brain at time for behavioral testing. *A* and *B*: in hyperammonemic (*A*) and PCS (*B*) rats, the total plasma concentrations of GR3027 are shown in μM. *C* and *D*: in hyperammonemic (*C*) and PCS (*D*) rats, the unbound brain concentrations of GR3027 are shown in nmol/kg. Note the similar exposures in the different rat models with the doses used, in hyperammonemic rats 3, 10, and 20 mg·kg^−1^·day^−1^ and in rats with PCS 0.7 and 2.5 mg·kg^−1^·day^−1^. Data are from the end of the study, i.e., after 9 wk of daily treatments with GR3027 in sesame oil given subcutaneously once daily. GAMSA, GABA_A_ receptor-modulating steroid antagonist.

In hyperammonemic rats the once-daily administration of GR3027 at 3, 10, and 20 mg/kg resulted in a dose-dependent exposure in plasma and brain. At the time of behavioral testing the total concentrations of GR3027 in plasma were 0.34 ± 0.03, 1.08 ± 0.11, and 1.95 ± 0.61 μM, respectively, and in the brain tissue the unbound concentrations of GR3027 were 6.1 ± 1.4, 11.6 ± 1.4, and 23 ± 5 nmol/kg, respectively (Fig. 7).

In PCS rats the exposures were also dose dependent, and with the lower doses used in these rats, 0.7 and 2.5 mg/kg, the exposures were very similar to those in the hyperammonemic rats. At the time of behavioral testing total concentrations in plasma were 0.48 ± 0.09 and 1.64 ± 0.30 μM, at 0.7 and 2.5 mg·kg^−1^·day^−1^, respectively, and unbound concentrations in the brain were 6.18 ± 0.97 and 17 ± 2 nmol/kg, respectively (Fig. 7).

These data clearly show that alteration of liver function or portosystemic shunts affects the systemic dose of the drug to be used to reach the levels obtained in the absence of liver failure. The data in Fig. 7 show that the levels of GR3027 in plasma and brain of PCS rats injected with 0.7 mg/kg are similar to those reached in hyperammonemic rats injected with 3 mg/kg. For PCS rats injected with 2.5 mg/kg, the levels reached in plasma are intermediate between those reached in hyperammonemic rats injected with 10 and 20 mg/kg. This indicates that doses in the range between four- and eightfold lower are required in PCS rats to reach the same plasma and brain GR3027 levels as in hyperammonemic rats without liver failure.

## DISCUSSION

We show for the first time that a selective inhibitor of positive neurosteroid modulation of the GABA_A_ receptor restores motor coordination and spatial learning and memory in rats with HE. GR3027 also partially restores the circadian rhythm of motor activity. Similar beneficial effects of GR3027 would be expected in the motor coordination and cognitive function of patients with HE.

Restoration of motor coordination by GR3027 would be due to a reduction of the GABAergic tone in cerebellum. Increased GABAergic tone in cerebellum induces motor incoordination, and the extracellular GABA concentration in cerebellum correlates with motor incoordination in rats (9). In hyperammonemic rats, extracellular GABA in cerebellum correlates with motor incoordination and reduction of GABA in cerebellum by pregnenolone sulfate restores motor coordination (17). Improved motor coordination is also achieved with the novel compound GR3027, which antagonizes the neurosteroids that enhance activation of GABA_A_ receptors.

We show that GR3027 restores spatial learning and memory in the radial and Morris water mazes in hyperammonemic and PCS rats. Impairment of learning and memory would be due to enhanced GABAergic tone in the hippocampus, and restoration by GR3027 would be due to reduction of the neurosteroid-induced activation of GABA_A_ receptors. Overactivation of GABA_A_ receptors in the hippocampus impairs spatial learning and memory in different situations (24, 33, 39, 41). Reducing the GABAergic tone improves spatial learning, for example, in models for Down syndrome, circadian arrhythmia, or alcohol ingestion (26, 31).

Enhanced activation of α5-containing GABA_A_ receptors during inflammation (41) or by etomidate (25) impairs memory, and α5-selective inverse agonists function as cognitive enhancers in animal models (32). Both α1-subunit- and α5-subunit-containing GABA_A_ receptors are involved in benzodiazepine-induced decreased learning and memory in the Morris water maze (33). Thus GR3027 antagonism of neurosteroid enhancement of both α1- and α5-containing hippocampal GABA_A_ receptors might be involved in restoration of learning and memory in HE. This agrees with our previous report showing that impairment of spatial learning in the Morris water maze by acute GABA_A_ receptor activation by neurosteroid treatment is improved by the neurosteroid antagonist UC1011 (39).

The results reported show that in PCS rats treatment with 0.7 mg/kg GR3027 is enough to restore motor coordination (Fig. 3*B*) but not spatial learning in the radial maze (Fig. 5*D*). This indicates that in PCS rats different doses of GR3027 are required to restore different types of neurological impairments. One reason for this could be that motor coordination is mainly modulated in the cerebellum while spatial learning is mainly modulated in the hippocampus. The mechanism of action of GR3027 is to antagonize the neurosteroid enhancement (allopregnanolone and THDOC) of GABA_A_ receptor activity. As the levels of these neurosteroids and the expression of GABA_A_ receptor subtypes are different in different brain areas (12, 45), different doses of GR3027 would be needed for modulation of each specific behavior.

GR3027 also partially improves the alterations in circadian rhythms of activity in rats with HE. The mechanisms underlying these alterations are not well known, and it would be speculative to discuss the mechanisms underlying the beneficial effects of GR3027. Nevertheless, altered circadian rhythms of activity are associated with altered sleep in PCS rats (23), which reproduce the sleep alterations of cirrhotic patients, who cannot sleep well during the night and show sleepiness during the day. The beneficial effect of GR3027 on circadian rhythms of activity suggests that it could also improve sleep in cirrhotic patients.

GR3027 is a GABA_A_ receptor-modulating steroid antagonist, GAMSA, that is, a compound that antagonizes the neurosteroid enhancement of GABA_A_ receptor activation but is without effect when activating steroids as allopregnanolone and THDOC are not present. Thus GR3027 does not antagonize the effect of GABA, which is preferable from a safety perspective as there will be no risk for seizure induction by GR3027. This is different from other GABA_A_ receptor active substances with beneficial effects on cognition in HE models (12, 17), as both pregnenolone sulfate and bicuculline block the effect of GABA at the receptor. Moreover, pregnenolone sulfate does not cross the blood-brain barrier and was administered intracerebrally (17), while GR3027 affords beneficial effects by peripheral administration. The action of GR3027 is also different from that of flumazenil, which transiently improves mental status in some HE patients (20). Flumazenil does not inhibit the effect of allopregnanolone at the GABA_A_ receptor but effectively antagonizes benzodiazepine effects (2).

The main advantage of GR3027 over currently used drugs for treating HE in patients is that GR3027 acts on a brain target, by modulating GABA_A_ receptor activation. Therefore GR3027 may normalize GABAergic neurotransmission and restore neurological functions altered because of overactivation of GABA_A_ receptors, even if hyperammonemia and inflammation associated with chronic liver disease remain present. Thus GR3027 may afford a more complete normalization of motor coordination and cognitive function by acting on a step that mediates the effects of hyperammonemia and inflammation on neurological functions.

## GRANTS

This study was financed by Umecrine Cognition AB.

## DISCLOSURES

This study was financed by Umecrine Cognition AB. All authors therefore have this conflict of interest.

## AUTHOR CONTRIBUTIONS

Author contributions: M.J., M.D., T.B., and V.F. conception and design of research; M.J., M.D., and V.F. interpreted results of experiments; M.J., M.D., T.B., and V.F. edited and revised manuscript; A.A., M.L., C.M., J.S., E.M., and G.R. performed experiments; A.A., M.L., C.M., J.S., E.M., G.R., and V.F. analyzed data; A.A. and M.L. prepared figures; M.D., T.B., and V.F. approved final version of manuscript; V.F. drafted manuscript.
